# Mechanistic and Clinical Evidence Supports a Key Role for Cell Division Cycle Associated 5 (CDCA5) as an Independent Predictor of Outcome in Invasive Breast Cancer

**DOI:** 10.3390/cancers14225643

**Published:** 2022-11-17

**Authors:** Yousif A. Kariri, Chitra Joseph, Mansour A. Alsaleem, Khloud A. Elsharawy, Sami Alsaeed, Michael S. Toss, Nigel P. Mongan, Andrew R. Green, Emad A. Rakha

**Affiliations:** 1 Academic Unit for Translational Medical Sciences, School of Medicine, Biodiscovery Institute, University Park Campus, University of Nottingham, Nottingham NG7 2RD, UK; 2 Department of Clinical Laboratory Science, Faculty of Applied Medical Science, Shaqra University, Shaqra 11961, Saudi Arabia; 3 Nottingham Breast Cancer Research Centre, Nottingham NG7 2RD, UK; 4 School of Medicine, Nottingham City Hospital, Nottingham University Hospitals NHS Trust and The University of Nottingham, Nottingham NG5 1PB, UK; 5 Department of Applied Medical Science, Applied College, Qassim University, Unayzah 56435, Saudi Arabia; 6 Department of Zoology, Faculty of Science, Damietta University, Damietta 34517, Egypt; 7 Department of Clinical Laboratory Science, Faculty of Applied Medical Sciences, Northern Border University, Arar 73244, Saudi Arabia; 8 Biodiscovery Institute, Faculty of Medicine and Health Sciences, School of Veterinary Medicine and Science, University of Nottingham, Nottingham NG7 2RD, UK; 9 Department of Pharmacology, Weill Cornell Medicine, New York, NY 10065, USA

**Keywords:** *CDCA5*, breast cancer, progression, prognosis

## Abstract

**Simple Summary:**

Breast cancer (BC) exhibits substantial genetic and clinical heterogeneity. Given the importance of understanding the molecular mechanisms underlying cancer cell division, migration, and evasion of apoptosis to develop novel therapies, identifying novel prognostic biomarkers is critical for accurate predictions of BC patient outcomes and treatment decisions. The goal of this retrospective study was to evaluate the potential prognostic value of the Cell Division Cycle Associated 5 (*CDCA5*), which is a member of the cyclin dependent kinase family and plays an important role in the phosphoinositide 3-kinase (PI3K)/AKT/mTOR signaling pathway involving in cell division, cancer cell migration, and apoptosis. Our findings emphasize the significance of *CDCA5* expression and its role in cell migration and evading apoptosis in BC tumor progression and worse patients’ clinical outcome. Further functional investigations are warranted to understand the crosstalk between cancer cell migration and evasion of apoptosis underlying mechanisms for targeted therapy development.

**Abstract:**

Background: Cell Division Cycle Associated 5 (*CDCA5*) plays a role in the phosphoinositide 3-kinase (PI3K)/AKT/mTOR signalling pathway involving cell division, cancer cell migration and apoptosis. This study aims to assess the prognostic and biological value of *CDCA5* in breast cancer (BC). Methods: The biological and prognostic value of *CDCA5* were evaluated at mRNA (*n* = 5109) and protein levels (*n* = 614) utilizing multiple well-characterized early stage BC cohorts. The effects of *CDCA5* knockdown (KD) on multiple oncogenic assays were assessed in vitro using a panel of BC cell lines. Results: this study examined cohorts showed that high *CDCA5* expression was correlated with features characteristic of aggressive behavior and poor prognosis, including the presence of high grade, large tumor size, lymphovascular invasion (LVI), hormone receptor negativity and HER2 positivity. High *CDCA5* expression, at both mRNA and protein levels, was associated with shorter BC-specific survival independent of other variables (*p* = 0.034, Hazard ratio (HR) = 1.6, 95% CI; 1.1–2.3). In line with the clinical data, in vitro models indicated that *CDCA5* depletion results in a marked decrease in BC cell invasion and migration abilities and a significant accumulation of the BC cells in the G2/M-phase. Conclusions: These results provide evidence that *CDCA5* plays an important role in BC development and metastasis and could be used as a potential biomarker to predict disease progression in BC.

## 1. Introduction

Invasive breast cancer (BC) evolution and progression involves multiple steps, including cancer cell proliferation, invasion and migration. However, the underlying molecular mechanisms and driver genes controlling BC cell behavior remain to be fully characterized [1,2,3]. Further investigation of the cellular machinery involved in the key biological processes can provide improved insights by identifying novel genes controlling BC progression and metastasis, which can eventually be used to develop new treatment strategies and improve patient outcomes.

The gene encoding the Cell Cycle Division Associated 5 (*CDCA5*) (also known as Sororin), is located on chromosome 11q12.1, a genomic region commonly altered in cancer [4,5]. *CDCA5* is a substrate of the anaphase-promoting complex and encodes 252 amino acids protein [6]. During BC angiogenesis, BC stem cell transcription factors and mammosphare formation (such as; tumor size and number of tumor spheroids) were reported to be significantly elevated as a results from the induced of *CDCA5* expression, which might highlight its role in tumor progression and development [7]. *CDCA5* along with the cyclin dependent kinase 1 (*CDK1*), is part of the cyclin dependent kinase family, plays an essential role in the separation of sister chromatids during the cell cycle S and G2/M phases [8,9]. An in vitro study in gastric cancer showed that *CDCA5* knockdown reduces cancer cell migration and promotes cancer cell apoptosis by inducing G2/M arrest [10]. *CDCA5* is also positively associated with other well-established cell cycle factors such as cell division cycle protein 2 (*CDC2*) and cyclin B1 [11] and it plays a key role in the phosphoinositide 3-kinase (PI3K)/AKT/mTOR signaling pathway, which contributes to tumor progression [11].

The upregulation of *CDCA5* expression occurs in several types of cancer, including hepatocellular carcinoma [12], colorectal [13], oral squamous cell carcinoma [14], implying that it may act as an oncogene promoting tumor progression. We have previously identified the novel role *CDCA5* as a strong predictor of LVI positivity in BC by using bioinformatic approaches to mine the publicly available trnnscriptomic BC cohort [15]. However, the role of *CDCA5* in BC LVI development and progression remains to be characterized. Therefore, this study aims to assess the expression of *CDCA5* in BC at the mRNA (METABRIC cohort) and protein levels (Nottingham BC cohort) to evaluate its association with clinicopathological parameters including LVI and BC patient outcome. In addition, it aims to investigate the impacts of *CDCA5* knockdown using in vitro models with relevant BC cell lines.

## 2. Materials and Methods

### 2.1. Transcriptomic Analysis

To explore *CDCA5* mRNA expression in BC, gene expression data were obtained from the TNM-plot (https://www.tnmplot.com/ accessed on 20 July 2021) and UALCAN (http://ualcan.path.uab.edu/index.html; accessed on 20 July 2021) datasets, which together include 1097 primary tumors 113 normal tissue samples, and 7 metastatic samples [16,17]. To validate the prognostic and molecular significance of *CDCA5* in BC, the online analytical module Kaplan–Meier Plotter (*n* = 2032) [18], and the Molecular Taxonomy of BC International Consortium (METABRIC) dataset (*n* = 1980) (Supplementary Table S1) [19] were used.

### 2.2. CDCA5 Immunohistochemistry

Prior to IHC staining, the validity of the mouse ploy colonel anti-*CDCA5* anti-body (HPA023691, Sigma-Aldrich, Gillingham, UK, 1:750/1 h) was checked using immunoblotting. The specificity of the *CDCA5* expression was validated using the SKBR3 and MDA MB-231 human BC cells (obtained from the American Type Culture Col-lection, Rockville, MD, USA). The rabbit β-actin antibody (clone AC-15, Sig-ma-Aldrich, Gillingham, UK) was used at 1:5000/1 h as a housekeeping protein and showed a band at approximately 42 KDa. A specific bands for the *CDCA5* protein expression were detected at the expected molecular weight after incubation overnight.

To evaluate the expression of *CDCA5* protein within BC tissue, 10 full-face BC tissue sections were selected based on different tumor grades and histological types. Tissue microarrays (TMA) Grand Master^®^ (3D HISTECH^®^, Budapest, Hungary) were used to array the tumor samples as previously described [20]. *CDCA5* immunohistochemistry was carried out using the Novolink Max Polymer Detection system (Leica, Newcastle, UK). Heat-induced citrate antigen retrieval (pH 6.0) was used and the *CDCA5* antibody incubated at room temperature for 1 h (dilution 1:25). The evaluation of the cytoplasmic staining for *CDCA5* in invasive tumor cells was performed using a modified histochemical score (H-score) [21]. TMA cores were only assessed if the invasive tumor burden was >15%. Scoring was conducted by an expert assessor (YK) and a subset of cases was independently scored (SA) to measure the interobserver concordance. The interclass correlation coefficient (ICC) concordance among both observers was 0.75, indicating excellent concordance. Normal kidney tissue was used as a positive tissue control (Figure S1A). A negative control omitting the primary antibody was carried out.

### 2.3. Immunohistochemical Analysis

To evaluate the expression of *CDCA5* protein, a well-characterized cohort of BC (*n* = 614) was used. The cohort characteristics are described in (Supplementary Table S1). The samples were collected from patients presenting at the Nottingham City Hospital NHS Trust as previously described [22]. The outcome data, including BC specific survival (BCSS) and distant metastasis free interval (DMFI), were defined as the time from the date of primary surgery until the time of the patients’ death due to BC or the occurrence of distant metastasis (DM), respectively.

To establish the prognostic value of *CDCA5* mRNA expression, the available data have been used to illustrate the *CDCA5* mRNA expression interaction with adhesion molecules, proliferation gene (*MKi-67*), matrix metalloproteinase markers (MMPs) (contributed during cancer cell progression, invasion, and metastasis), PI3K/AKT/mTOR pathway, apoptosis and cyclin-related genes in the METABRIC cohort. Furthermore, the protein interaction role and the influence of *CDCA5* expression in relation to the prognostic markers (p53 and Ki67), adhesion markers (E-cadherin (*CDH1*) and N-cadherin (*CDH2*)), epidermal growth factor receptor (EGFR), basal-phenotype, as well as cyclin E and PI3K were included in this study as per previous publications [23,24,25].

### 2.4. Evaluation of the Functional Activity of CDCA5 in BC Cell Lines

BC cell lines were selected according to the Western blot results, the protein and mRNA results, which presented high expression of *CDCA5* in HER2 + SKBR3 cell line and MDA-MB-231 TNBC cell lines. Both cells were obtained from the American Type Culture Collection (ATCC, Manassas, VA, USA) and cultured as recommended by ATCC, 10% Foetal Bovine Serum (10% FBS) was added to RPMI 1640 medium during the cultivation of MDA MB-231. SK-BR-3 was cultured in McCoy’s 5A medium that had been modified with L-glutamine and sodium bicarbonate liquid (M9309; Sigma, UK) in addition to 10% FBS. All cells were tested to confirm absence of mycoplasma (CUL001B; R&D Systems, Abingdon, UK) during experiments. Cells were maintained in a humidified incubator at 37 °C in a 5% CO_2_ environment.

### 2.5. Transient (siRNA) Knockdowns (KD) of CDCA5

To investigate possible functional consequences of *CDCA5* depletion and study its role in BC survival efficiency, proliferation, invasion, migration and cell cycle processes, we used an siRNA-based approach in BC cell lines. Differential protein expression of *CDCA5* in BC presented high expression in SKBR3 (HER2+) and MDA MB-231 (TNBC) cell lines). We have tested three siRNA (IDs: 129005, 129006, and 129007) and all siRNA targeting *CDCA5* showed a similar knockdown effect on *CDCA5* protein expression using MDA MB-231 cell lines (Figure S1D). Accordingly, we used only one siRNA (ID 129005) for subsequent functional studies.

The forward transfection of the siRNA procedure was followed according to the manufacturer’s instructions. In summary, SKBR3 and MDA MB-231 were seeded in a 6-well plate at a cell density of 3 × 10^5^ cells per well and incubated overnight. The following day, the cells reached approximately 40% confluence and were transfected with *CDCA5* siRNA (ID 129005), the validation siRNA of *CDCA5* (ID 129006), and scrambled negative control siRNA (Cat# 4390843), purchased from ThermoFisher Scientific, UK at 30 nM concentration for SKBR3 and 10 nM for MDA MB-231. siRNAs were delivered to the cells in OptiMEM medium (ThermoFisher, Cat#: 31985062, Dorset, UK) using lipofectamine RNAiMAX reagent (ThermoFisher, Cat#: 13778075, Vilnius, Lithuania). RIPA buffer (89900; ThermoFisher Scientific, Loughborough, UK), was used to collect the cell lysate and the efficiency of transfection was detected using Western blotting [26]. All experiments were carried out in triplicate.

### 2.6. Phenotypic and Mechanistic Characterisation of CDCA5 Depletion

The effect of *CDCA5* depletion, on cancer cell proliferation was determined using CellTitre Aqueous One MTS Solution Cell Proliferation Assay obtained from Promega. In brief, cells were seeded at 3000 per cells/well in a 96-well plate in the incubator at 37 °C in a 5% CO_2_ atmosphere. The proliferative ability was detected at time point zero (T0), 24 h (T24) and 48 h (T48) hours by adding MTS reagent as suggested from the manufacturer’s protocol. The plate was incubated for 1 h and absorbance of cells was measured using a Synergy™ 4 (BioTek Instruments, Winooski, VT, USA) at 490 nm.

The effect of *CDCA5* depletion on cell invasion was measured using the CytoSelect 24-Well Cell Invasion Assay (Basement Membrane, Colorimetric) from Cell Biolabs. Prior to detaching and seeding the cells in matrix-coated trans-wells, the cells were incubated overnight in serum free media. The cells were treated with media containing 10% FBS for 24 h in the original wells to enhance the cells’ ability of invasion. The cells in the top of the trans-well were removed and the bottom cells were treated with cell stain for 10 min, before the extraction bottom-cells using an extraction solution; the invasion ability was detected at 560 nm as per the manufacturer’s protocol.

For clonogenic assays, 32 cells/cm^2^ were seeded in 6-well plates and incubated at 37 °C in a 5% CO_2_ atmosphere for 14 days. After 14 days, the plate was washed with PBS, fixed using 70% ethanol and stained using 0.5% crystal violet stain. Colonies with ≥50 cells were counted manually and the surviving fraction was calculated [27].

A wound healing assay was performed using Culture-Insert 2 Well, in µ-Dish^35 mm^ (Thistle Scientific, Glasgow, UK) according to the manufacturer’s instructions. Briefly, 1.5 × 10^5^ cells in 10% FBS media were seeded and incubated overnight at 37 °C in a 5% CO_2_ atmosphere. The following day, a confluent layer of appropriate cell attachment was performed, then the well wall was removed gently by using sterile tweezers. The cells were washed using 1% FBS cultured media to focus only on the cell migratory behaviour. After washing, the cells were incubated for at least 1 h. The wound images were taken using an inverted microscope (LEICA DMI3000B, Leica microsystems, Wetzlar, Germany) in T0, T24 h and T48 h. Image J software (1.52 version, National Institutes of Health, Bethesda, MD, USA) was used to measure the cell migration area, and the percentage of wound closure was calculated.

The effect of *CDCA5* depletion on cell cycle was assessed using flow cytometry. To this end 2.5 × 10^5^ cells per well were seeded in 6-well plates overnight. Cells were collected by trypsinisation and washed with cold-icy PBS and then fixed using 70% ethanol for at least 4 h. After the samples were centrifuged for 5 min at 7000 rpm to remove the fixative solution and cells were stained using a 1x mixture of PBS, Propidium Iodide stain and RNase (ab 1394718, Abcam, Cambridge, UK) as recommended by the manufacturer. Following incubation at 37 °C in the dark for 30 min, samples were analysed on a MACSQuant^®^ analyser flow cytometer (Miltenyi Biotec, Bergisch Gladbach, Germany) and the data were analysed using FlowJo software (version 14.0.0.0., Ashland, OR, USA).

### 2.7. Statistical Analysis

For the clinical study, SPSS (Version 28.0, IBM SPSS Statistic, Chicago, IL, USA) was used to perform the statistical analysis. The median was used to determine the cut-off point (8.35, 990/1980) in order to categorise the mRNA METABRIC data into high and low subgroups. For the *CDCA5* protein, the median was used as the cut-off point which categorized the expression into low (H-score < 30) and high (H score ≥ 30, 284/614) expressions, respectively. The Pearson correlation coefficient test was used to assess the correlation between *CDCA5* mRNA and other related genes. The association between the clinical-pathological features and *CDCA5* protein expression was assessed using the Chi-square test. The prognostic significance of the *CDCA5* expression was measured using the Kaplan–Meier survival curves. The multivariate survival analysis was evaluated using the Cox proportional hazard method. The statistical significance of the clinical-pathological factors and survival was defined by a *p*-value < 0.05 (two-tailed). This study followed the reporting recommendations for tumor markers prognostic studies (REMARK) criteria [28].

Data analysis for in vitro experiments was performed on GraphPad Prism software (version 5, San Diego, CA, USA). Student’s T-tests analysis was used to measure the differences between the siRNA scrambled and siRNA-*CDCA5*. All experiments were presented as means ± standard error of mean (SEM) of three independent experiments. The *p*-values * ≤ 0.05, ** ≤ 0.01, *** ≤ 0.001 and **** ≤ 0.0001 were considered statistically significant.

## 3. Results

### 3.1. Significance of CDCA5 mRNA Expression in BC

**Figure 1:**
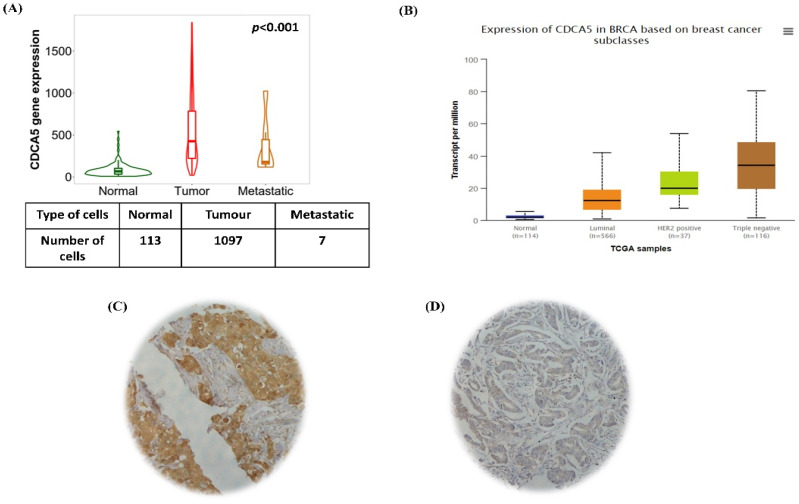
Distribution of mRNA and immunohistochemical expression of *CDCA5* in BC. (**A**) RNA seq dataset comprising 1097 breast tumoral samples, 113 adjunct normal tissue and 7 metastatic samples were analyzed to evaluate *CDCA5* expression using TNM data portal. (**B**) Evaluation of *CDCA5* expression using PAM50 classification for BC obtained from UALCAN data portal. Representative immunohistochemical *CDCA5* protein expression in invasive BC cores (power 20×). (**C**) *CDCA5* strong cytoplasmic staining of invasive BC cells and (**D**) *CDCA5* weak cytoplasmic staining of invasive BC cells.

Within the TNM-plotter BC dataset, high *CDCA5* mRNA expression was documented in BC tissues compared to normal breast samples (Figure 1A). When molecular BC subtypes were considered in the UALCAN dataset, *CDCA5* high expression was seen in the HER2-enriched and TNBC molecular classes more than the luminal A (estrogen receptor (ER)-positive/progesterone receptor (PR)-positive (ER+/PR+) class (Figure 1B).

In the METABRIC cohort, high *CDCA5* mRNA expression demonstrated a significant correlation with well-established poor prognosis characteristics including larger tumor size, high histological grade, nodal status-positivity, LVI-positivity, ER & PR negativity, and HER2-positivity (*p* < 0.001, Table 1). Regarding histological tumor subtype, high *CDCA5* mRNA expression was associated with ductal carcinoma of no special type (NST) compared to the special subtypes (*p* < 0.001; Table 1).

The correlations between *CDCA5* expression and other functionally related biomarkers at the METABRIC cohort are illustrated in Table 2. High *CDCA5* mRNA expression was positively associated with the expression of cell cycle related genes, including *CDKN2A*, *CCNA1*, *CCNA2*, *CCNB1*, *CCNB2*, *CCND3*, *CCNE1*, *CCNE2*, *CCNT1*, *CDK1*, *CDK2*, *CDK4*, *CDK5* and *CDK6* (*p* < 0.05. Table 2). Furthermore, high *CDCA5* mRNA expression was positively associated with the expression was positively correlated with PI3K/AKT/mTOR pathway and apoptosis related genes, including *PIK3CA*, *AKT1*, *MTOR BAX*, and *MYC* (*p* < 0.05. Table 2). Additionally, high mRNA expression of *CDCA5* was positively correlated with proliferation gene (*MKi-67*) (*p* < 0.001; Table 2). Regarding adhesion molecules, high mRNA expression of *CDCA5* was correlated with a higher expression of *CDH2*, however it was negatively correlated with *CDH1* expression (all *p* < 0.001; Table 2).

**Figure 2:**
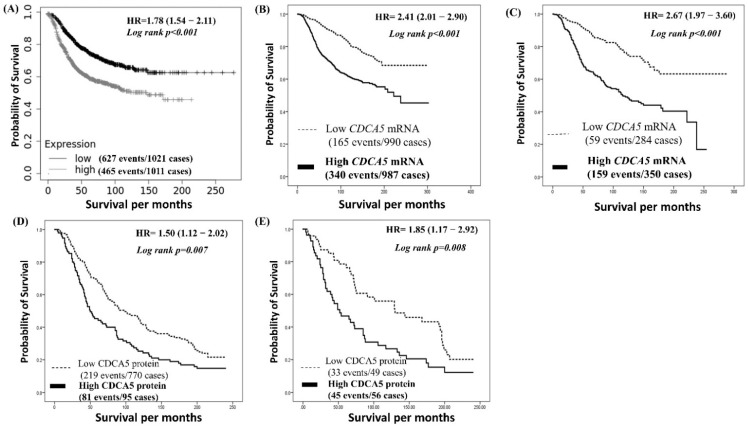
Patient outcomes of BC survival on the METABRIC and the Nottingham BC cohorts. (**A**) Cumulative survival of BC patients stratified by *CDCA5* mRNA expression in the KM-Plotter cohort. (**B**) Cumulative survival of BC patients stratified by *CDCA5* mRNA expression in breast tumors in METABRIC. (**C**) Cumulative survival of BC patients stratified by *CDCA5* mRNA expression in LVI-positive BC in METABRIC. (**D**) Cumulative survival of BC stratified by CDCA5 protein expression. (**E**) Cumulative survival of BC patients stratified by CDCA5 protein expression in the Nottingham LVI-positive cohort.

Outcome analysis using the KM plotter datasets revealed that BC patients who had a tumor with high *CDCA5* mRNA expression had a worse survival rate compared with those who had low *CDCA5* mRNA expression (*p* < 0.001, Hazard ratio (HR) 1.78, 95% CI; 1.52–2.07; Figure 2A). Similarly, outcome analysis using METABRIC cohort showed a positive association between *CDCA5* mRNA expression and shorter survival (*p* < 0.001, HR 2.41, 95% CI; 2.01–2.90; Figure 2B). When examining the prognostic significance of CDCA5 mRNA expression in various molecular subtypes, our results showed that luminal A (*p* < 0.001; Figure S2A), Luminal B (*p* = 0.005; Figure S2B), and Normal like (*p* = 0.002; Figure S2C) BC patients with high *CDCA5 mRNA* expression had significant association with poor patients’ survival compared to patients who had low *CDCA5 mRNA* expression but not TNBC nor HER2+ classes. Additionally, our multivariate analysis showed that *CDCA5* mRNA expression was an independent prognostic marker associated with worse BCSS (*p* < 0.001, HR 1.70, 95% CI: 1.30–2.22). This association was independent of other established prognostic factors: LVI, tumor size, histological grade, nodal stage ER, PR, and HER2 status (Table 3).

Due to the strong correlation between *CDCA5* and LVI, the METABRIC cohort was stratified based on LVI status. This showed that high expression of *CDCA5* mRNA is a strong induction of a shorter BCSS in the LVI-positive subgroup (*p* < 0.001, HR, 2.67, 95% CI; 1.97–3.60; Figure 2C). High *CDCA5* expression in the LVI-negative subgroup survival was not statistically significant in terms of outcome (*p* = 0.284, HR 1.34, 95% CI; 0.78–2.30; Figure S1B).

### 3.2. CDCA5 Protein Expression

Full-face tissue sections showed a homogeneous cytoplasmic expression in the BC tissue which was higher than normal breast tissue. A total of 284/614 (46%) cases showed high cytoplasmic *CDCA5* expression (Figure 1C) while low expression was seen in 330/614 cases (Figure 1D). High *CDCA5* expression was significantly associated with features characteristic of aggressive behaviour and poor prognosis including younger age at presentation, high histological grade, lymph node positivity, the poor prognosis group of Nottingham Prognostic Index (NPI), LVI-positivity, ER&PR negativity and HER2-positivity (*p* < 0.05; Table 4). When the BC cohort was stratified based on the BC-molecular subtypes, high expression of *CDCA5* protein was significantly associated with the HER2+ enriched BC-subtype (*p* = 0.001, Table 4).

The association between *CDCA5* expression and other functionally related biomarkers was sought at the protein level where a high *CDCA5* protein expression was positively correlated with a higher expression of cell cycle related markers including cyclin E (*p* < 0.001), p53 (*p* < 0.001), Ki-67 (*p* < 0.001), basal-phenotype (*p* = 0.010), and EGFR (*p* = 0.040) (Table 3). There was a negative correlation between *CDCA5* and E-cadherin expression (*p* = 0.033; Table 4). A trend towards a positive correlation with PI3K was observed, however it remained of no statistical significance (Table 4).

Patients with high *CDCA5* expression had significantly worse overall BCSS (*p* = 0.007, HR 1.50, 95% CI; 1.12–2.02; Figure 2D) compared to patients who had a low *CDCA5* expression. Multivariate analysis showed that *CDCA5* expression was an independent prognostic marker associated with worse BCSS (*p* = 0.044, HR 1.42, 95% CI: 1.01–2.01). This association was independent of other established prognostic factors: LVI, tumor size, histological grade, nodal stage, ER, PR, and HER2 status (Table 3). In the same vein as to the mRNA analysis, when the BC cohort was stratified based on LVI, the high expression of the *CDCA5* protein was associated with shorter BCSS in the LVI-positive subgroup (*p* = 0.008, HR 1.85, 95% CI; 1.17–2.92; Figure 2E). High *CDCA5* expression in LVI-negative tumors did not predict survival (*p* = 0.726, HR 1.10, 95% CI; 0.66–1.80; Figure S1C).

### 3.3. In Vitro Investigation of CDCA5

**Figure 3:**
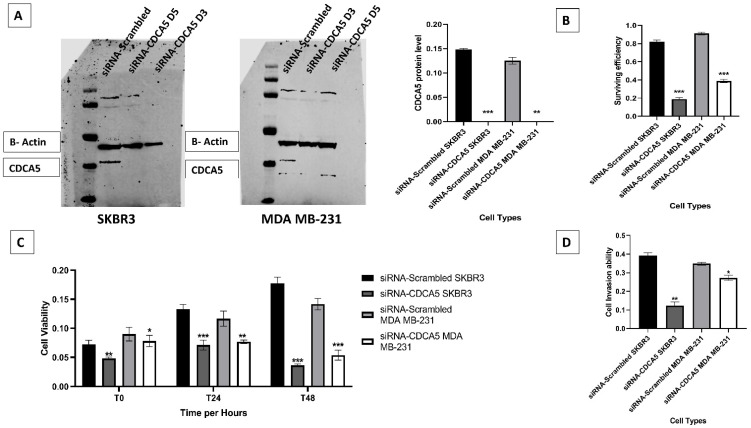
The protein expression of *CDCA5* was evaluated in HER2-enriched (SKBR3) and TNBC (MDA MB-231) cell lines. (**A**) Western blot of *CDCA5* levels in SKBR3 and MDA MB-231 BC cell lines transfected with *CDCA5* siRNA and scrambled siRNA control. (**B**) Clonogenic survival assay for SKBR3, MDA MB-231 control and knockdown cells (**C**) Proliferation ability for SKBR3 and MDA MB-231 control and knockdown was evaluated using MTS assay. (**D**) Invasion assay quantification of SKBR3 & MDA MB-231 control and knockdown cells. *p* values are indicated as follows; ‘*’ *p* < 0.05, ‘**’ *p* < 0.01, ‘***’ *p* < 0.001. Error bar indicates standard error of the mean (SEM).

After the clinical findings and the evidence of the prognostic value of *CDCA5* in BC, we decide to confirm its role in the key biological process using in vitro models. *CDCA5* depletion reduced HER2-enriched SKBR3 and TNBC MDA MB-231 cells lines (*p* < 0.0001 and *p* = 0.006, respectively; Figure 3A).

### 3.4. CDCA5 Promotes Cell Survival Efficiency, Proliferation and Invasion Ability

Following the clinical findings and the evidence of the prognostic value of *CDCA5* in BC, we sought to confirm it role in the key biological process using in vitro models. Consistent with a role for *CDCA5* in cell survival, proliferation, and invasion, *CDCA5* depletion impaired these processes in both SKBR3 and TNBC MDA MB-231 cells lines (*p* < 0.0001 and *p* = 0.006, respectively; Figure 3A). In concordance with *CDCA5* protein data expression, *CDCA5* knockdown significantly increased cell survival ability in both cell models (all *p* < 0.0001; Figure 3B) as compared to the scrambled control cells. *CDCA5* knockdown significantly reduced cell proliferative (all *p* < 0.0001, Figure 3C) and invasion ability of both SKBR3 and MDA MB-231 cells (*p* = 0.004 and *p* = 0.021, respectively; Figure 3D).

### 3.5. CDCA5 Increases Cell Migration and Cell Cycle Ability

**Figure 4:**
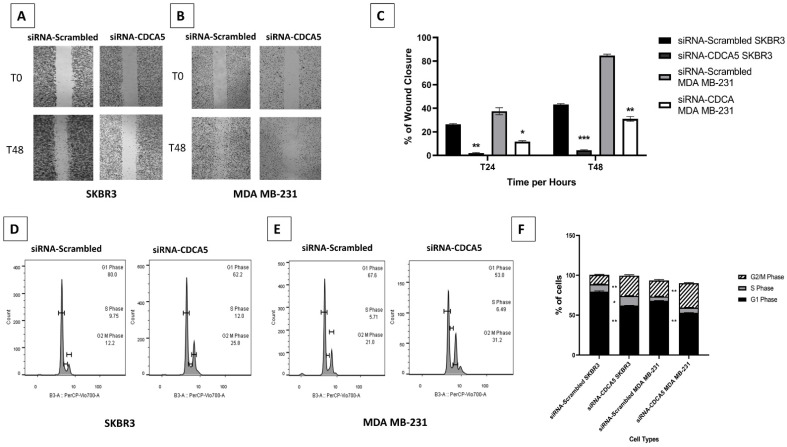
*CDCA5* cancer cell migration and cell cycle result. (**A**–**C**), Representative images of wound closure assay in SKBR3 & MDA-MB 231 BC cell lines transfected with scrambled siRNA control and *CDCA5* siRNA, wound measuring was performed in ImageJ software. (**D**–**F**). Representative images and quantification of cell cycle progression by flow cytometry in the SKBR3 and MDA MB-231 BC cell lines transfected with *CDCA5* siRNA compared to control scrambled cells. *p* values are indicated as follows; ‘*’ *p* < 0.05, ‘**’ *p* < 0.01, ‘***’ *p* < 0.001. Error bar indicates standard error of the mean (SEM).

*CDCA5* depletion showed a significant decrease in cell wound closure in both SKBR3 and MDA MB-231 (*p* < 0.0001 and *p* = 0.003, respectively, Figure 4A–C) compared to controls. In both SKBR3 and MDA MB-231, *CDCA5* knockdown impaired a significant reduction in G1-phase (*p* = 0.003 and 0.002, respectively) alongside a significant accumulation in G2/M-phase (*p* = 0.004 and 0.008, respectively) (Figure 4D–F).

## 4. Discussion

The cell cycle is a series of complex events where a cell seeks to accurately duplicate its molecular content and divides into two daughter cells. This process is crucial to cellular and tissue homeostasis. Several factors can modify and indeed impair cell cycle, including aberrant expression of oncogenes, tumor suppressor genes, cyclin proteins and cyclin-dependent kinases [29]. The functional alteration of key cell cycle regulators is an important contributor to carcinogenesis. *CDCA5* expression, which plays an essential role in the cell cycle, has been identified as an upregulated gene in various types of cancer, including breast [30], bladder [11], esophageal squamous cell carcinoma [31], colorectal [13] and hepatocellular cancer [12]. Similarly, a study by Phan and colleagues suggested the association between *CDCA5* overexpression and progression of BC [30]. However, the role of *CDCA5* expression in LVI, a key determinant of poor outcomes, has yet to be defined.

The current study indicates that there is a significant association between high *CDCA5* and aggressive tumor features including larger tumor size, high tumor grade, LVI positivity, hormonal receptor negativity, HER2 positivity and independent prognostic factor for worse patient outcomes. These results are consistent with the previous studies [11,12,13,30,31] which report that *CDCA5* is significantly correlated with cancer progression. Furthermore, the significant association with nodal status and high *CDCA5* expression at both the mRNA and protein level indicates its ability to be involved in BC invasion and metastasis which was confirmed by the in vivo and in vitro models in hepatocellular carcinoma [32] and in this study in BC.

The mRNA expression of *CDCA5* showed a positive association with cyclin-related markers which play a critical role in the cell cycle process (G1/S) and cell proliferation [33]. Consistent with *CDCA5* mRNA expression, *CDCA5* protein expression showed a significant positive association with Cyclin E1 which is well-known as a critical factor that promotes G1/S transition while functioning as an oncogene in BC [34]. Our study speculated that endogenous *CDCA5* may enhance the probability of tumor oncogenesis in the examined cell lines. In accordance with this study findings, high *CDCA5* expression may contribute to tumor proliferation via regulating cyclin E1 expression [35]. These results are consistent with the results of a previous study performed on gastric cancer, which showed that the high expression of cyclin E1 may resume the proliferation ability and enhance the G1-phase arrest in vivo [36]. Therefore, high expression of *CDCA5* could play a significant role in tumor progression, invasion, and metastasis via cyclin E1.

*CDCA5* showed differential expression with cadherins. It was negatively correlated with E cadherin (*CDH1*), although it showed a positive correlation with N cadherin (*CDH2*). Based on our observation, high *CDCA5* expression may contribute to suppressing the cell adhesion process by facilitating BC tumor cell migration through the lymphatic vessels and by invasion through activating the Wnt and PI3K signalling pathways [37].

In addition, we have demonstrated that the high protein expression of *CDCA5* was positively associated with EGFR, which plays important roles in mechanisms contributing to cellular migration and invasion [38]. Together, *CDCA5* may have an important role in the upregulation of N-cadherin and EGFR and concomitant downregulation in E-cadherin is a key step in BC progression and is associated with that activation of the β-catenin; LEF/TCF regulation of vimentin expression, which has previously been shown to promote BC invasion and metastasis [3,39]. The negative correlation between *CDCA5* and E cadherin could also be complementary, reducing the mechanisms enhancing cell migration and invasion which may suggest the role of *CDCA5* in LVI. High protein expression of *CDCA5* was an independent prognostic marker for poorer patient survival. Among the BC subgroups, high expression of *CDCA5* was recognized to elevate in HER2-enriched BC. The HER2-positive BC type is one of the most aggressive types of BC that is strongly associated with cancer cell adhesion [40,41]. Furthermore, several studies were reported stromal tumor-infiltrating lymphocytes as potent prognostics and predictive biomarkers for HER2-positive BC [26,42,43]. The results of our study may identify the promising role of *CDCA5* in inflammatory BC. Furthermore, the functional assessment of *CDCA5* in BC is warranted to evaluate its role in cancer cell adhesion pathways.

Furthermore, as evinced in our study, silencing of *CDCA5* expression revealed a significant reduction in migratory and invasive capabilities in BC cell lines, showing the potential of *CDCA5* to regulate cancer cell migration among aggressive cancer types. Likewise, a study by Rezaei and colleagues proposed that the increased expression of N-cadherin enhances the production of MMPs to prepare a suitable environment for cancer cell migration by degrading the basement membrane at the primary site to simplify the migration process [44]. Nonetheless, this study also showed that high *CDCA5* expression was significantly associated with an increase in cell cycle activity markers such as Ki67 and Cyclin B1. This in consistent with a previous study by Ji et al., which indicated that high *CDCA5* expression is associated with poor survival in prostate cancer [45] Furthermore, our functional results suggest that decreasing *CDCA5* occurs with an accumulation of cells in the G2/M phase of cell cycle in aggressive tumors. This may be explained by pro-oncogenic role of *CDCA5* during the cell cycle that impairs cancer apoptosis while promoting cell proliferation via the PI3K/AKT/mTOR signalling pathway, which was shown on our results at transcriptomic level. This is merely a potential mechanistic speculation as the correlation at mRNA level may not imply that expression levels of *CDCA5* are associated with higher activation of these pathways. Further phospho-protein analysis of PI3K/AKT/mTOR mediators between control and *CDCA5* knockdown cells is warranted to confirm this association and the involvement of *CDCA5* in the pathway activation. However, a previous study conducted on bladder cancer showed similar association between *CDCA5* and PI3K/AKT/mTOR pathway, which supports our hypothesis. [11]. The strong association with cadherins, TGF-β1 and LVI positivity strengthens our in vitro findings, highlighting the importance of *CDCA5* in BC tumor progression and further supports its role in promoting the migratory and invasive mechanisms.

While this study presents interesting findings given at both the mRNA and protein levels that support the critical role of *CDCA5* in BC, we acknowledge some limitations. Firstly, this study uses a retrospective cohort. Accordingly, a well-characterized randomized clinical trial accompanied by a uniform treatment type and new TMA cohorts is recommended for an independent evaluation of the expression of *CDCA5* in BC. However, we have highlighted the role of *CDCA5* in BC using several internal and external cohorts in this study, regardless of our TMA having been collected in retrospect. Secondly, further in vivo and in vitro functional studies are warranted in order to identify the exact molecular mechanism(s) underlying the *CDCA5* models and to confirm its ability as a therapeutic potential factor in BC-LVI.

## 5. Conclusions

In conclusion, the high expression of *CDCA5* in BC is associated with LVI-positivity and worse prognostic parameters. It is an independent prognostic marker for shorter patient survival. *CDCA5* appears to play a significant role in cancer cell proliferation, migration, invasion and metastasis. Further functional studies which evaluate the molecular mechanisms underlying the *CDCA5* models and its therapeutic potential would be merited.

## Figures and Tables

**Table 1:** Association of *CDCA5* mRNA expression with clinicopathological characteristics in the METABRIC (*n* = 1980).

Parameters	METABRIC Cohort		
	Low *CDCA5*	High *CDCA5*	*p* Value
	*N* (%)	*N* (%)	
Tumor size			
≤2.0cm	482 (49)	501 (51)	**<0.001**
>2.0cm	377 (39)	**600 (61)**	
Nodal Status			
Negative	582 (56)	404 (44)	**<0.001**
Positive	453 (43)	**534 (57)**	
Histological Grade			
Grade 1and 2	675 (72)	265 (28)	**<0.001**
Grade 3	265 (27)	**696 (73)**	
Tumor Histological subtypes			
Ductal NST	691 (45)	**853 (55)**	**<0.001**
Lobular	27 (84)	5 (16)	
Medullary-like	155 (76)	48 (24)	
Special type	105 (71)	42 (29)	
Lymphovascular Invasion			
Negative	494 (64)	284 (36)	**<0.001**
Positive	436 (55)	**351 (45)**	
Estrogen receptor			
Negative	88 (9)	**902 (91)**	**<0.001**
Positive	386 (39)	604 (61)	
Progesterone receptor			
Negative	310 (31)	**680 (69)**	**<0.001**
Positive	630 (64)	360 (36)	
Human epidermal growth factor receptor 2			
Negative	107 (95)	140 (5)	**<0.001**
Positive	883 (80)	**850 (20)**	
EGFR			
Negative	515 (52)	475 (48%)	0.080
Positive	475 (48)	**515 (52%)**	

Abbreviations: METABRIC, The Molecular Taxonomy of Breast Cancer International Consortium. Significant correlations are in bold.

**Table 2:** Correlation of high *CDCA5* mRNA expression with mRNA expression of adhesion molecule, MMPs, proliferation, PI3K/AKT/mTOR pathway, apoptosis, and cyclin related genes.

Gene Names	METABRIC Cohort	
	Correlation Value	*p* Value
Adhesion molecule genes		
*CDH1*	−0.100	**<0.001**
*CDH2*	0.163	**<0.001**
	Proliferation gene	
*MKi-67*	0.689	**<0.001**
MMPs related genes		
*MMP7*	0.180	**<0.001**
*MMP9*	0.324	**<0.001**
*MMP11*	0.097	**<0.001**
*MMP12*	0.354	**<0.001**
*MMP14*	0.086	**<0.001**
*MMP15*	0.230	**<0.001**
*MMP20*	0.145	**<0.001**
*MMP25*	0.138	**<0.001**
	PI3K/AKT/mTOR pathway genes	
*PIK3CD*	0.173	**<0.001**
*AKT1*	0.052	**0.022**
*MTOR*	0.125	**<0.001**
	Apoptosis gens	
*BAX*	0.270	**<0.001**
*MYC*	0.173	**<0.001**
Cyclin related genes		
*CDKN2A*	0.396	**<0.001**
*CCNA1*	0.236	**<0.001**
*CCNA2*	0.838	**<0.001**
*CCNB1*	0.614	**<0.001**
*CCNB2*	0.883	**<0.001**
*CCND3*	0.096	**<0.001**
*CCNE1*	0.680	**<0.001**
*CCNE2*	0.671	**<0.001**
*CCNT1*	0.128	**<0.001**
*CDK1*	0.726	**<0.001**
*CDK2*	0.572	**<0.001**
*CDK4*	0.442	**<0.001**
*CDK5*	0.191	**<0.001**
*CDK6*	0.221	**<0.001**

Significant correlations are in bold.

**Table 3:** Multivariate Cox proportional hazard regression analysis for predictors of BCSS in the METABRIC (*n* = 1980) and Nottingham BC cohort (*n* = 614).

Factors	BCSS in METABRIC Cohort			BCSS in Nottingham BC Cohort		
	Hazard Ratio	95% CI	*p* Value	Hazard Ratio	95% CI	*p* Value
*CDCA5*	1.70	1.30–2.22	**<0.001**	1.42	1.01–2.01	**0.044**
Tumor size	1.52	1.19–1.95	**<0.001**	1.51	1.03–2.20	**0.034**
Tumor grade	1.06	0.81–1.40	0.669	1.73	1.19–2.51	**0.004**
Tumor Stage	2.11	1.54–2.90	**<0.001**	1.60	1.26–2.03	**<0.001**
LVI	1.84	1.46–2.33	**<0.001**	1.37	0.97–1.96	0.078
ER	0.89	0.67–1.20	0.440	0.83	0.51–1.35	0.453
PR	0.77	0.58–1.01	0.055	0.62	0.34–0.97	**0.038**
HER2 status	1.57	1.17–2.10	**0.002**	1.18	0.76–1.83	0.469

Significant correlations are in bold.

**Table 4:** Association between *CDCA5* protein expression and clinicopathological characteristics of the studies cohort (*n* = 614).

Parameters	*CDCA5* Protein Expression		
	Low *N* (%)	High *N* (%)	*p* Value
Tumor size			
≤2.0cm	172 (58)	126 (42)	0.060
>2.0cm	158 (50)	157 (50)	
Nodal Status			
Negative	189 (53)	165 (47)	**0.005**
Positive	123 (48)	**133 (52)**	
Histological Grade			
Grade 1	49 (68)	23 (32)	**<0.001**
Grade 2	128 (64)	73 (36)	
Grade 3	154 (45)	**187 (55)**	
Tumor Histological Subtypes			
Ductal NST	131 (46)	**85 (33)**	**<0.001**
Lobular	90 (30)	40 (16)	
Medullary	33 (12)	80 (31)	
Special type	33 (12)	51 (20)	
Lymphovascular invasion			
Negative	196 (57)	146 (43)	**0.004**
Positive	84 (44)	**106 (56)**	
Nottingham prognostic index			
Good prognostic group	102(65)	55(35)	**0.005**
Moderate prognostic group	167(50)	166(50)	
Poor prognostic group	59(50)	60(50)	
Age			
<50	116 (49)	**121 (51)**	**0.049**
>50	212 (57)	160 (43)	
Estrogen Receptor			
Negative	51 (31)	**115 (69)**	**0.040**
Positive	276 (62)	168 (38)	
Progesterone Receptor			
Negative	105 (41)	**152 (59)**	**0.001**
Positive	214(63)	128 (37)	
Human epidermal growth factor receptor 2			
Negative	287 (56)	223 (44)	**0.004**
Positive	33 (39)	**51 (61)**	
P53			
Negative	235 (58)	170 (42)	**0.001**
Positive	80 (42)	**111 (58)**	
Ki67			
Negative	114(63)	67(37)	**0.001**
Positive	153(47)	**171(53)**	
Epidermal growth factor receptor (EGFR)			
Negative	264 (56)	208 (44)	**0.040**
Positive	59 (46)	**70 (54)**	
E-cadherin			
Negative	199 (51)	**193 (49)**	**0.033**
Positive	122(60)	82 (40)	
N-Cadherin			
Negative	69 (55)	56 (45)	0.684
Positive	151 (39)	238 (61)	
Basal phenotype			
Negative	361 (79)	96 (21)	**0.010**
Positive	246 (71)	**102 (29)**	
Cyclin E			
Negative	81 (62)	50 (38)	**<0.001**
Positive	9 (26)	**25 (74)**	
Phosphoinositide 3-kinase			
Negative	56 (59)	46 (41)	0.215
Positive	184 (52)	**171 (48)**	
IHC-Subtypes			
Luminal A	131 (62)	80 (38)	**0.001**
Luminal B	90 (62)	40 (31)	
Her2 enriched	33 (28)	**103 (72)**	
TNBC	28 (39)	51 (61)	

Significant correlations are in bold.

## Data Availability

The authors confirm the data that has been used in this work is available on reasonable request.

## Supplementary Materials

The following supporting information can be downloaded at: https://www.mdpi.com/article/10.3390/cancers14225643/s1, Figure S1: LVI negative patient BC survival outcomes stratified by the *CDCA5* expression at the transcriptomic and proteomic levels and *CDCA5* protein knockdown day three using different siRNAs in the MDA-MB-231 BC cell line. (A) Cumulative survival of BC patients stratified by *CDCA5* mRNA expression in the LVI- METABRIC cohort. (B) Cumulative survival of BC patients stratified by *CDCA5* protein expression in the LVI- Nottingham cohort. (C) *CDCA5* protein expression in human kidney tissue (positive control). D) Representing the *CDCA5* protein expression knockdown for MDA-MB-231 using three siRNAs (s129003, 129006 and 129007) and control scrambled cells. Chart illustrating the differences in the *CDCA5* protein expression between the three siRNAs and the control scrambled cells.; Figure S2: Molecular subtypes patient BC survival outcomes stratified by the *CDCA5* expression at the transcriptomic level. (A) Cumu-lative survival of BC patients stratified by *CDCA5* mRNA expression in the Luminal-A BC cohort. (B) Cumulative survival of BC patients stratified by *CDCA5* mRNA expression in the Luminal-B BC cohort. (C) Cumulative survival of BC patients stratified by *CDCA5* mRNA expression in the normal-like BC cohort.; Table S1: Clinicopathological parameters of the Molecular Taxonomy of Breast Cancer International Consortium (METABRIC) and Nottingham validation series.

## References

[1] Aleskandarany M.A., Sonbul S.N., Mukherjee A., Rakha E.A. (2015). Molecular Mechanisms Underlying Lymphovascular Invasion in Invasive Breast Cancer. Pathobiology.

[2] Rakha E.A., Martin S., Lee A.H.S., Morgan D., Pharoah P.D.P., Hodi Z., Macmillan D., Ellis I.O. (2012). The prognostic significance of lymphovascular invasion in invasive breast carcinoma. Cancer.

[3] Kariri Y.A., Aleskandarany M.A., Joseph C., Kurozumi S., Mohammed O.J., Toss M.S., Green A.R., Rakha E.A. (2020). Molecular Complexity of Lymphovascular Invasion: The Role of Cell Migration in Breast Cancer as a Prototype. Pathobiology.

[4] Vijai J., Kirchhoff T., Schrader K.A., Brown J., Dutra-Clarke A.V., Manschreck C., Hansen N., Rau-Murthy R., Sarrel K., Przybylo J. (2013). Susceptibility loci associated with specific and shared subtypes of lymphoid malignancies. PLoS Genet..

[5] Johanneson B., Deutsch K., McIntosh L., Friedrichsen-Karyadi D.M., Janer M., Kwon E.M., Iwasaki L., Hood L., Ostrander E.A., Stanford J.L. (2007). Suggestive genetic linkage to chromosome 11p11.2-q12.2 in hereditary prostate cancer families with primary kidney cancer. Prostate.

[6] Nishiyama T., Ladurner R., Schmitz J., Kreidl E., Schleiffer A., Bhaskara V., Bando M., Shirahige K., Hyman A.A., Mechtler K. (2010). Sororin mediates sister chromatid cohesion by antagonizing Wapl. Cell.

[7] Hu H., Xiang Y., Zhang X.Y., Deng Y., Wan F.J., Huang Y., Liao X.H., Zhang T.C. (2022). CDCA5 promotes the progression of breast cancer and serves as a potential prognostic biomarker. Oncol. Rep..

[8] Borton M.T., Rashid M.S., Dreier M.R., Taylor W.R. (2016). Multiple Levels of Regulation of Sororin by Cdk1 and Aurora B. J. Cell. Biochem..

[9] Schmitz J., Watrin E., Lenart P., Mechtler K., Peters J.M. (2007). Sororin is required for stable binding of cohesin to chromatin and for sister chromatid cohesion in interphase. Curr. Biol..

[10] Chen T., Huang Z., Tian Y., Wang H., Ouyang P., Chen H., Wu L., Lin B., He R. (2017). Role of triosephosphate isomerase and downstream functional genes on gastric cancer. Oncol. Rep..

[11] Fu G., Xu Z., Chen X., Pan H., Wang Y., Jin B. (2020). CDCA5 functions as a tumor promoter in bladder cancer by dysregulating mitochondria-mediated apoptosis, cell cycle regulation and PI3k/AKT/mTOR pathway activation. J. Cancer.

[12] Chen H., Chen J., Zhao L., Song W., Xuan Z., Chen J., Li Z., Song G., Hong L., Song P. (2019). CDCA5, Transcribed by E2F1, Promotes Oncogenesis by Enhancing Cell Proliferation and Inhibiting Apoptosis via the AKT Pathway in Hepatocellular Carcinoma. J. Cancer.

[13] Shen A., Liu L., Chen H., Qi F., Huang Y., Lin J., Sferra T.J., Sankararaman S., Wei L., Chu J. (2019). Cell division cycle associated 5 promotes colorectal cancer progression by activating the ERK signaling pathway. Oncogenesis.

[14] Tokuzen N., Nakashiro K., Tanaka H., Iwamoto K., Hamakawa H. (2016). Therapeutic potential of targeting cell division cycle associated 5 for oral squamous cell carcinoma. Oncotarget.

[15] Kurozumi S., Joseph C., Sonbul S., Alsaeed S., Kariri Y., Aljohani A., Raafat S., Alsaleem M., Ogden A., Johnston S.J. (2019). A key genomic subtype associated with lymphovascular invasion in invasive breast cancer. Br. J. Cancer.

[16] Bartha Á., Győrffy B. (2020). TNMplot.com: A web tool for the comparison of gene expression in normal, tumor and metastatic tissues. BioRxiv.

[17] Chandrashekar D.S., Bashel B., Balasubramanya S.A.H., Creighton C.J., Ponce-Rodriguez I., Chakravarthi B., Varambally S. (2017). UALCAN: A Portal for Facilitating Tumor Subgroup Gene Expression and Survival Analyses. Neoplasia.

[18] Gyorffy B., Lanczky A., Eklund A.C., Denkert C., Budczies J., Li Q., Szallasi Z. (2010). An online survival analysis tool to rapidly assess the effect of 22,277 genes on breast cancer prognosis using microarray data of 1809 patients. Breast Cancer Res. Treat..

[19] Curtis C., Shah S.P., Chin S.F., Turashvili G., Rueda O.M., Dunning M.J., Speed D., Lynch A.G., Samarajiwa S., Yuan Y. (2012). The genomic and transcriptomic architecture of 2000 breast tumours reveals novel subgroups. Nature.

[20] Abd El-Rehim D.M., Ball G., Pinder S.E., Rakha E., Paish C., Robertson J.F., Macmillan D., Blamey R.W., Ellis I.O. (2005). High-throughput protein expression analysis using tissue microarray technology of a large well-characterised series identifies biologically distinct classes of breast cancer confirming recent cDNA expression analyses. Int. J. Cancer.

[21] McCarty K.S., McCarty K.S. (1984). Histochemical approaches to steroid receptor analyses. Semin. Diagn. Pathol..

[22] Kariri Y.A., Joseph C., Kurozumi S., Toss M.S., Alsaleem M., Raafat S., Mongan N.P., Aleskandarany M.A., Green A.R., Rakha E.A. (2019). Prognostic significance of KN motif and ankyrin repeat domains 1 (KANK1) in invasive breast cancer. Breast Cancer Res. Treat..

[23] Muftah A.A., Aleskandarany M.A., Al-Kaabi M.M., Sonbul S.N., Diez-Rodriguez M., Nolan C.C., Caldas C., Ellis I.O., Rakha E.A., Green A.R. (2017). Ki67 expression in invasive breast cancer: The use of tissue microarrays compared with whole tissue sections. Breast Cancer Res. Treat..

[24] Rolland P., Spendlove I., Madjd Z., Rakha E.A., Patel P., Ellis I.O., Durrant L. (2007). The p53 positive Bcl-2 negative phenotype is an independent marker of prognosis in breast cancer. Int. J. Cancer.

[25] Rakha E.A., Abd El Rehim D., Pinder S.E., Lewis S.A., Ellis I.O. (2005). E-cadherin expression in invasive non-lobular carcinoma of the breast and its prognostic significance. Histopathology.

[26] Kariri Y.A., Alsaleem M., Joseph C., Alsaeed S., Aljohani A., Shiino S., Mohammed O.J., Toss M.S., Green A.R., Rakha E.A. (2020). The prognostic significance of interferon-stimulated gene 15 (ISG15) in invasive breast cancer. Breast Cancer Res. Treat..

[27] Franken N.A., Rodermond H.M., Stap J., Haveman J., van Bree C. (2006). Clonogenic assay of cells in vitro. Nat. Protoc..

[28] Sauerbrei W., Taube S.E., McShane L.M., Cavenagh M.M., Altman D.G. (2018). Reporting Recommendations for Tumor Marker Prognostic Studies (REMARK): An Abridged Explanation and Elaboration. J. Natl. Cancer Inst..

[29] Wenzel E.S., Singh A.T.K. (2018). Cell-cycle Checkpoints and Aneuploidy on the Path to Cancer. In Vivo.

[30] Phan N.N., Wang C.Y., Li K.L., Chen C.F., Chiao C.C., Yu H.G., Huang P.L., Lin Y.C. (2018). Distinct expression of CDCA3, CDCA5, and CDCA8 leads to shorter relapse free survival in breast cancer patient. Oncotarget.

[31] Xu J., Zhu C., Yu Y., Wu W., Cao J., Li Z., Dai J., Wang C., Tang Y., Zhu Q. (2019). Systematic cancer-testis gene expression analysis identified CDCA5 as a potential therapeutic target in esophageal squamous cell carcinoma. EBioMedicine.

[32] Tian Y., Wu J., Chagas C., Du Y., Lyu H., He Y., Qi S., Peng Y., Hu J. (2018). CDCA5 overexpression is an Indicator of poor prognosis in patients with hepatocellular carcinoma (HCC). BMC Cancer.

[33] Noguchi T., Dobashi Y., Minehara H., Itoman M., Kameya T. (2000). Involvement of cyclins in cell proliferation and their clinical implications in soft tissue smooth muscle tumors. Am. J. Pathol..

[34] Karakas C., Biernacka A., Bui T., Sahin A.A., Yi M., Akli S., Schafer J., Alexander A., Adjapong O., Hunt K.K. (2016). Cytoplasmic Cyclin E and Phospho-Cyclin-Dependent Kinase 2 Are Biomarkers of Aggressive Breast Cancer. Am. J. Pathol..

[35] Luhtala S., Staff S., Tanner M., Isola J. (2016). Cyclin E amplification, over-expression, and relapse-free survival in HER-2-positive primary breast cancer. Tumour Biol..

[36] Zhang Z., Shen M., Zhou G. (2018). Upregulation of CDCA5 promotes gastric cancer malignant progression via influencing cyclin E1. Biochem. Biophys. Res. Commun..

[37] Alsaleem M., Toss M.S., Joseph C., Aleskandarany M., Kurozumi S., Alshankyty I., Ogden A., Rida P.C.G., Ellis I.O., Aneja R. (2019). The molecular mechanisms underlying reduced E-cadherin expression in invasive ductal carcinoma of the breast: High throughput analysis of large cohorts. Mod. Pathol..

[38] Masuda H., Zhang D., Bartholomeusz C., Doihara H., Hortobagyi G.N., Ueno N.T. (2012). Role of epidermal growth factor receptor in breast cancer. Breast Cancer Res. Treat..

[39] Gilles C., Polette M., Mestdagt M., Nawrocki-Raby B., Ruggeri P., Birembaut P., Foidart J.M. (2003). Transactivation of vimentin by beta-catenin in human breast cancer cells. Cancer Res..

[40] Schade B., Lesurf R., Sanguin-Gendreau V., Bui T., Deblois G., O’Toole S.A., Millar E.K., Zardawi S.J., Lopez-Knowles E., Sutherland R.L. (2013). β-Catenin signaling is a critical event in ErbB2-mediated mammary tumor progression. Cancer Res..

[41] Falchook G.S., Moulder S.L., Wheler J.J., Jiang Y., Bastida C.C., Kurzrock R. (2013). Dual HER2 inhibition in combination with anti-VEGF treatment is active in heavily pretreated HER2-positive breast cancer. Ann. Oncol..

[42] Savas P., Salgado R., Denkert C., Sotiriou C., Darcy P.K., Smyth M.J., Loi S. (2016). Clinical relevance of host immunity in breast cancer: From TILs to the clinic. Nat. Rev. Clin. Oncol..

[43] Hammerl D., Smid M., Timmermans A.M., Sleijfer S., Martens J.W.M., Debets R. (2018). Breast cancer genomics and immuno-oncological markers to guide immune therapies. Semin. Cancer Biol..

[44] Rezaei M., Friedrich K., Wielockx B., Kuzmanov A., Kettelhake A., Labelle M., Schnittler H., Baretton G., Breier G. (2012). Interplay between neural-cadherin and vascular endothelial-cadherin in breast cancer progression. Breast Cancer Res..

[45] Ji J., Shen T., Li Y., Liu Y., Shang Z., Niu Y. (2021). CDCA5 promotes the progression of prostate cancer by affecting the ERK signalling pathway. Oncol. Rep..

