# Supplementary material for: Mechanistic and Clinical Evidence Supports a Key Role for Cell Division Cycle Associated 5 (CDCA5) as an Independent Predictor of Outcome in Invasive Breast Cancer
Source: Cancers (Basel). 2022 Nov 17;14(22):5643. doi: 10.3390/cancers14225643 (PMC9688237; doi:10.3390/cancers14225643)
Supplement: Supplementary file 1 [file cancers-14-05643-s001.zip › cancers-1886924-supplementary.pdf]

cancers

MDPI

Supplementals

Mechanistic and Clinical Evidence Supports a Key Role for Cell Division Cycle Associated 5 (CDCA5) as an Independent Predictor of Outcome in Invasive Breast Cancer

Yousif A. Kariri [1,2,3], Chitra Joseph [4], Mansour A. Alsaleem [1,3,5], Khloud A. Elsharawy [1,3,6], Sami Alsaeed [1,3,7], Michael S. Toss [3,4], Nigel P. Mongan [8,9], Andrew R. Green [1,3] and Emad A. Rakha [1,3,4,*]

Table S1. Clinicopathological parameters of the Molecular Taxonomy of Breast Cancer International Consortium (METABRIC) and Nottingham validation series.

Pathological parameters	METABRIC series N (%)	Nottingham set N (%)
**Age**		
< 50 years	424 (21.4)	469 (30.5)
≥ 50 years	1426 (78.6)	1070 (69.5)
**Tumor size**		
< 2cm	623(31.8)	939 (61.1)
≥ 2cm	1337(68.2)	599 (38.9)
**Grade**		
1	169 (9.0)	231 (15.0)
2	770 (40.7)	622 (40.4)
3	952 (50.3)	685 (44.5)
**Tumour types**		
Ductal (including mixed)	1545 (83.6)	1335 (86.9)
Lobular	148 (8.0)	120 (7.8)
Medullary-like	32 (1.7)	13 (0.8)
Miscellaneous	12 (0.6)	9 (0.6)
Special type	113 (6.1)	60 (3.9)
**Vascular invasion**		
Definite	Not available	451 (29.3)
Negative/Probable		1086 (70.7)
**Lymph Node Stage**		
1	1035 (52.5)	955 (62.2)
2	623 (31.5)	428 (27.9)
3	315 (16.0)	153 (10.0)
**Estrogen receptor**		
Negative	472 (23.8)	300 (19.5)
Positive	1508 (76.2)	1240 (80.5)
**Progesterone receptor**		
Negative	938 (47.4)	612 (41.8)
Positive	1042 (52.6)	853 (58.2)
**Human epidermal growth factor receptor 2**		
Negative	1734 (87.5)	1376 (89.9)
Positive	246 (12.5)	155 (10.1)

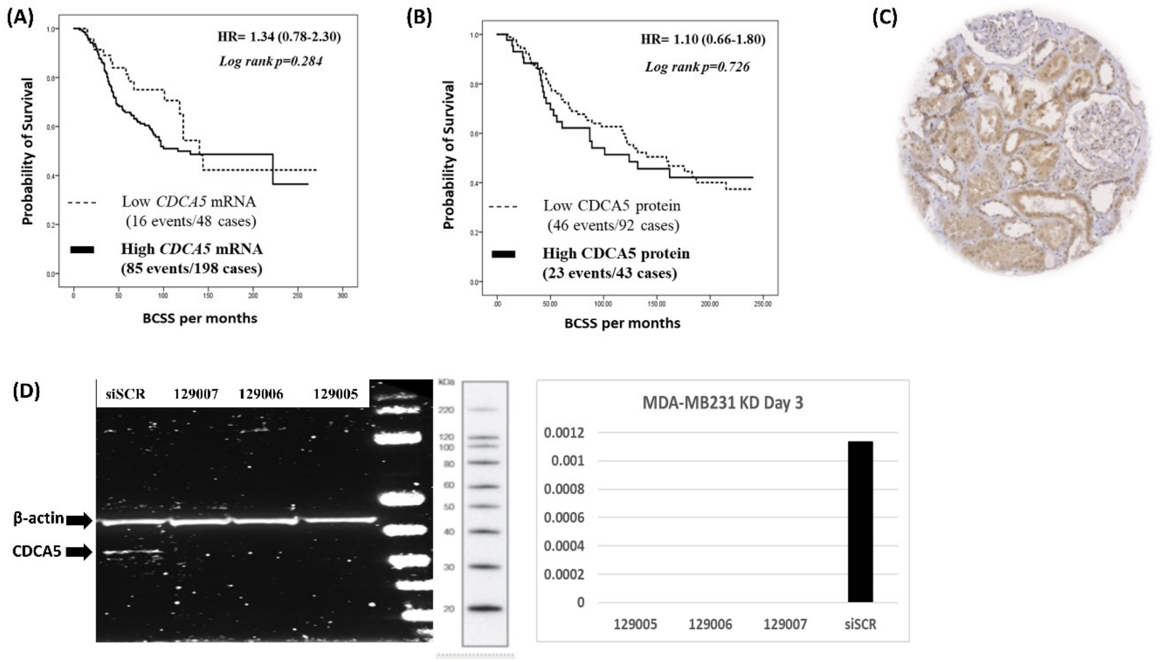

Figure S1. LVI negative patient BC survival outcomes stratified by the CDCA5 expression at the transcriptomic and proteomic levels and CDCA5 protein knockdown day three using different siRNAs in the MDA-MB-231 BC cell line. (A) Cumulative survival of BC patients stratified by *CDCA5* mRNA expression in the LVI- METABRIC cohort. (B) Cumulative survival of BC patients stratified by CDCA5 protein expression in the LVI- Nottingham cohort. (C) CDCA5 protein expression in human kidney tissue (positive control). D) Representing the CDCA5 protein expression knockdown for MDA-MB-231 using three siRNAs (s129003, 129006 and 129007) and control scrambled cells. Chart illustrating the differences in the CDCA5 protein expression between the three siRNAs and the control scrambled cells.

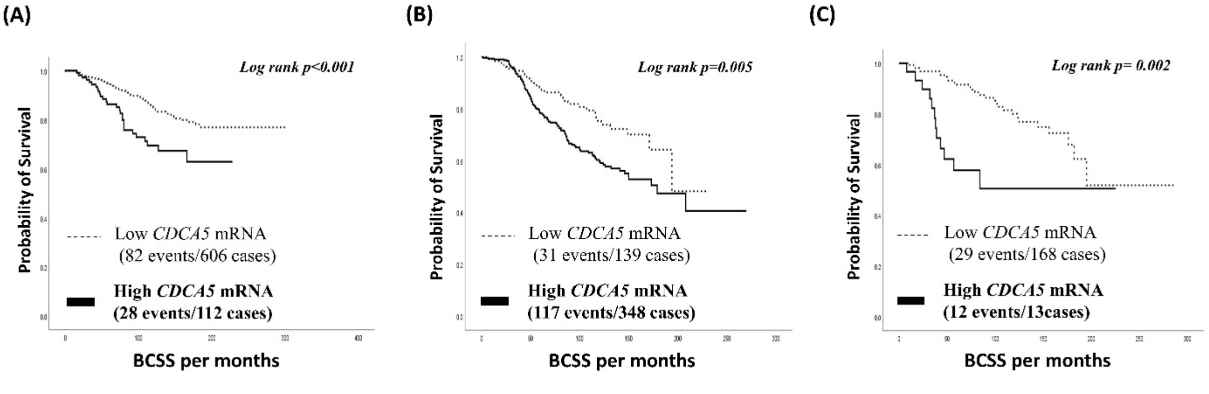

Figure S2. Molecular subtypes patient BC survival outcomes stratified by the CDCA5 expression at the transcriptomic level. (A) Cumulative survival of BC patients stratified by *CDCA5* mRNA expression in the Luminal-A BC cohort. (B) Cumulative survival of BC patients stratified by *CDCA5* mRNA expression in the Luminal-B BC cohort. (C) Cumulative survival of BC patients stratified by *CDCA5* mRNA expression in the normal-like BC cohort.
